# Mutually Dependent Clustering of SynDIG4/PRRT1 and AMPA Receptor Subunits GluA1 and GluA2 in Heterologous Cells and Primary Neurons

**DOI:** 10.3389/fnmol.2022.788620

**Published:** 2022-04-08

**Authors:** Kristopher E. Plambeck, Chun-Wei He, Hector H. Navarro, Elva Díaz

**Affiliations:** Department of Pharmacology, University of California Davis School of Medicine, Davis, CA, United States

**Keywords:** AMPA receptor (AMPAR), SynDIG4, PRRT1, SynDIG1, synaptic plasticity

## Abstract

Regulation of α-amino-3-hydroxy-5-methyl-4-isoxazolepropionic acid (AMPA)-type glutamate receptors (AMPARs) at synapses is a predominant mechanism for regulating synaptic strength. We identified the transmembrane protein synapse differentiation-induced gene 1 (SynDIG1; SD1) as an AMPAR interacting protein that regulates excitatory synaptic strength and AMPAR number both *in vitro* and *in vivo.* The related protein SynDIG4 (SD4; also known as PRRT1) was identified in several independent proteomic screens in complex with AMPARs, suggesting that it may function as an AMPAR auxiliary factor. Here, we show that the co-expression of SD4 with GluA1 or GluA2 homomeric AMPARs in COS cells leads to a 50 or 33% increase in the mean area of AMPAR puncta, respectively. This effect is accentuated when AMPAR puncta are stratified for co-localization with SD4, resulting in a 100 and 65% increase in GluA1 and GluA2 puncta, respectively. Chimeric proteins expressing only the membrane bound domain of SD4 co-expressed with full-length GluA1 or GluA2 recapitulated the effects of wild-type (WT) SD4. Additionally, the mean puncta area of GluA1 or GluA2 chimeras expressing the membrane and C-terminal domains increased significantly when co-localized with WT SD4. Similarly, the co-expression of GluA1 or GluA2 with SD4 results in a significant increase in the mean area of SD4 puncta co-localized with GluA1 or GluA2, respectively. Last, we observed a significant increase in the co-localization of SD4 with GluA1 after glycine induced long-term potentiation (LTP). The mean size of GluA1 puncta was significantly increased when stratified, indicating that co-localization with SD4 increases synaptic GluA1 cluster size during LTP. These data indicate mutually dependent clustering of SD4 and AMPAR subunits both in COS cells and primary hippocampal neurons, suggesting a mechanism for increased synaptic strength during chemical LTP.

## Introduction

Neurons form precise connections known as synapses that are necessary for cell–cell communication. During excitatory synapse development, pre-synaptic axon terminals responsible for the export of signaling molecules pair with post-synaptic dendritic spines that contain glutamate receptors, scaffolding molecules, and cytoskeletal elements (McAllister, 2007). At excitatory synapses, there are two types of ionotropic glutamate receptors which are recruited to the synaptic site *via* different mechanisms (Lissin et al., 1998): N-methyl-D-aspartate receptors (NMDARs) and α-amino-3-hydroxy-5-methyl-4-isoxazolepropionic acid receptors (AMPARs). NMDARs are first recruited to the dendritic surface during early maturation of excitatory synapses while the later recruitment of AMPARs stabilize the synapse and represent a mature synaptic structure (Scheiffele, 2003). AMPARs are necessary for fast synaptic transmission, and changes in the number of synaptic AMPARs directly reflect changes in synaptic strength (Huganir and Nicoll, 2013). Previous studies have identified a diverse group of AMPAR interacting proteins necessary for the modulation of AMPAR biophysical properties and trafficking to the synapse (Díaz, 2010; Jackson and Nicoll, 2011; Jacobi and von Engelhardt, 2017). For example, the AMPAR auxiliary protein Stargazin, a member of the transmembrane AMPAR regulating protein (TARP) family TARP-γ2, has been observed to decrease the deactivation and desensitization rates of AMPARs, as well as increase forward trafficking of AMPARs to the cell surface (Chen et al., 2000). Stargazin/TARP-γ2 influences AMPARs through interaction with two distinct protein domains (Tomita et al., 2003, 2005), of which the transmembrane (TM) domains TM3 and TM4 and extracellular loop 2 of stargazin/TARP-γ2 have been found to be critically important (Ben-Yaacov et al., 2017). Additional AMPAR auxiliary proteins, such as the Cornichons (CNIHs) (Schwenk et al., 2009) and cysteine-knot AMPAR modulating proteins (CKAMPs) (von Engelhardt et al., 2010) have been shown to affect the functional properties of AMPARs. Therefore, AMPAR localization and channel properties are regulated by a wide array of distinct molecules.

The brain-specific type II transmembrane protein synapse differentiation-induced gene 1 (SynDIG1; SD1) was previously identified as an AMPAR interacting protein which regulates excitatory synapse development (Kalashnikova et al., 2010). Specifically, SD1 clusters with AMPARs in heterologous cells and modulates the number of functional GluA1 and GluA2 containing AMPARs at excitatory synapses. The knockdown of SD1 results in a decrease in the number and strength of excitatory synapses. However, SD1 does not affect the biophysical properties of AMPARs, such as deactivation and desensitization to glutamate (Lovero et al., 2013), indicating SD1 is not a typical auxiliary factor.

Synapse differentiation-induced gene 1 (SynDIG4; SD4), also known as proline-rich transmembrane protein 1 (PRRT1), was identified by three independent proteomic studies (von Engelhardt et al., 2010; Schwenk et al., 2012; Shanks et al., 2012) and demonstrates sequence similarity to SD1 (Kalashnikova et al., 2010). Surprisingly, SD4 is de-enriched at the post-synaptic density (PSD) and co-localizes with the AMPAR subunit GluA1 at extra-synaptic sites in primary neurons (Kirk et al., 2016), implying a role of SD4 outside of the PSD. SD4 has been shown to modify AMPAR gating kinetics in a subunit-dependent manner (Matt et al., 2018). Specifically, SD4 slows the deactivation of GluA1 homomers, as well as GluA1/A2 heteromeric AMPARs. Additionally, SD4 slows the desensitization of GluA1 homomers but not GluA1/A2 heteromers. Interestingly, these effects are potentiated when expressed with TARP-γ8 (Matt et al., 2018), indicating that SD4 may function in AMPAR complexes containing TARP-γ8. In support of this conclusion, a recent cryo-electron microscopy (cryo-EM) study demonstrated that SD4 is associated with native AMPAR complexes that contain both TARP-γ8 and CNIH-2 (Yu et al., 2021).

The primary goal of this study is to further elucidate the role of SD4 in regulating GluA1- and GluA2-containing AMPARs using a structure-function approach. The link between AMPAR subunits and SD4 is necessary to establish a mechanism by which SD4 may affect the localization and trafficking of AMPARs important for synaptic plasticity in the brain. We hypothesize that SD4 is necessary for establishing a reserve pool of AMPARs important for synaptic plasticity through its ability to cluster AMPARs at extra-synaptic sites. The present study identifies the regions sufficient for the clustering of SD4 and the AMPAR subunits GluA1 and GluA2 in heterologous COS cells. Intriguingly, the colocalization of SD4 and AMPAR subunits indicates mutually dependent clustering of AMPAR subunits and SD4, respectively. This observation is recapitulated in primary hippocampal neurons, suggesting a mechanism by which SD4 establishes a reserve pool of extrasynaptic AMPARs that can be employed for SD4-dependent synaptic plasticity.

## Materials and Methods

### Antibodies

The following antibodies were used: mouse IgG1 anti-GluA1 [Neuromab; Cat# 75-327; RRID: AB_2315840; Immunocytochemistry (ICC) 1:200; Immunoblotting (IB) 1:2,000]; mouse IgG2a anti-SynDIG4 (NeuroMab; Cat# 73-409; RRID: AB_2491106; ICC 1:200; IB 1:2,000); mouse IgG2a anti-SynDIG1 (NeuroMab; Cat# 75-251; RRID: AB_10999753; ICC 1:200); rabbit anti- Interferon-induced transmembrane protein 3 (IFITM3) (ProteinTech; ICC 1:200; IB 1:2,000); rat anti-hemagglutinin (HA) (Roche; ICC 1:50; IB 1:1,000); guinea pig anti-vGlut1 (EMD Millipore; ICC 1:500); Alexa 488-conjugated anti-mouse IgG2a (Molecular Probes; ICC 1:200); Alexa 594-conjugated anti-rat (Jackson ImmunoResearch; ICC 1:200); Alexa 555-cross adsorbed anti-mouse IgG1 (Invitrogen; ICC 1:500); Alexa 649-conjugated anti-guinea pig (Jackson ImmunoResearch: ICC 1:500); mouse anti-beta tubulin (MilliporeSigma; Clone: AA2; IB 1:5,000); goat horseradish peroxidase (HRP) conjugated anti-rat (Invitrogen; IB 1:5,000); and goat HRP-conjugated anti-mouse (Invitrogen; IB 1:10,000).

### Constructs

A full length version of rat SD4 coding sequence was amplified by PCR from pHM6 expression vector and subcloned into pRK5 vector backbone provided by our collaborator Dr. Yael Stern-Bach at the Hebrew University of Jerusalem, Jerusalem, Israel. A full length version of mouse SD1 was expressed using a previously generated pHM6 construct (Kalashnikova et al., 2010). pCMV-HA-mIFITM3 was a gift from Howard Hang & Jacob Yount (Addgene plasmid # 58389; http://n2t.net/addgene:58389; RRID: Addgene_58389). Full length mouse IFITM3 was obtained from AddGene (#58389). SD4/IFITM3 chimeras (Table 1) were generated by sequential PCR amplification using megaprimers. Full length wild-type (WT) GluA1 was provided from the Stern-Bach lab and subcloned from the pGEM vector to the pRK5 expression vector. DNA vectors expressing full length GluA2 and GluK2, as well as the GluK2/A1 (Table 2, chimera #2–4) and GluK2/A2 le 3, chimera #1 and 2) chimeras, were additionally provided by the Stern-Bach lab. Additional GluK2/A1 (Table 2, chimeras #1, 5, and 6) and GluK2/A2 (Table 3, chimera #3) constructs were generated by sequential PCR amplification using the megaprimer method. All constructs contain an in-frame HA tag at the N-terminus for detection. Tables 1–3 identify the amino acid (a.a.) sequences of the indicated protein expressed within each chimeric molecule.

**TABLE 1:** Synapse differentiation-induced gene 4 (SD4)/IFITM3 chimeras.

HA-IFITM3/SD4	IFITM3	SD4
1) HA-IF-NTD/SD4-M	a.a. 1–59	a.a. 224–306
2) HA-SD4-NTD/IF-M	a.a. 60–137	a.a. 1–223

**TABLE 2:** GluK2/GluA1 chimeras.

HA-K2/A1	GluK2	GluA1
1) M1-3,S2,M4,CT	a.a. 1–561	a.a. 537–907
2) M1-3,M4,CT	a.a. 1–561; a.a. 660–819	a.a. 537–631; a.a. 806–907
3) M1-3, CT	a.a. 1–561; a.a. 660–840	a.a. 537–631; a.a. 827–907
4) M1-3	a.a. 1–561; a.a. 660–908	a.a. 537–631
5) S2,M4,CT	a.a. 1–659	a.a. 632–907
6) M4,CT	a.a. 1–819	a.a. 806–907

**TABLE 3:** GluK2/GluA2 chimeras.

HA-K2/A2	GluK2	GluA2
1) M1-3,S2,M4,CT	a.a. 1–561	a.a. 543–883
2) M1-3,S2,M4	a.a. 1–561; a.a. 841–908	a.a. 543–838
3) M4,CT	a.a. 1–819	a.a. 810–883

### Cell Culture

#### COS Cells

The primate cell culture line COS-7 (ATCC CRL-1651) was used for all experiments in heterologous cells. COS cells were grown in COS media containing DMEM (Life Technologies) supplemented with 10% Fetal Bovine Serum (Fisher Scientific) and 1% Penicillin/Streptomycin (Life Technologies). Cells were cultured at 37^°^C with 5% CO_2_.

#### Primary Neurons

Dissociated cultures of primary hippocampal neurons were generated from embryonic day 18 (E18) rat embryos as previously described (Kalashnikova et al., 2010). Cultures used an astrocyte feeder layer derived from rat cortex and grown to 70–90% confluency in 6-well plates in astrocyte plating medium (APM) containing 1X MEM, 10% donor horse serum, 0.6% glucose, and 5 ml pen/strep. Prior to hippocampal dissection, coverslips were treated with 1 M nitric acid and sterilized. Coverslips were then coated with 1 mg/ml poly-L-lysine (PLL) diluted in distilled water and incubated overnight at 37^°^C. After incubation, coverslips were washed 3 times with distilled water. Dissociated neurons were first cultured on coverslips in Neuronal Plating Media (NPM) containing 1X MEM, 10% donor horse serum, 0.45% glucose, 5 ml sodium pyruvate, and 5 ml pen/strep. After 6 h, neurons on coverslips were transferred to the astrocyte feeder layer that had been changed to Neuronal Maintenance Media (NMM) containing 1X neurobasal, 10 ml Glutamax, 5 ml sodium pyruvate, and 5 ml pen/strep. After 4 days, the anti-mitotic AraC was added at a final concentration of 5 μM. A half volume change of the NMM was performed every 5 days. Neurons were utilized at approximately days *in vitro* (DIV) 12–14 depending on confluency and maturity.

### Immunoblotting

COS cells were seeded in 6-well plates at a density of 300,000 cells per well in COS media. Transfection was performed with 2 μg of DNA using Lipofectamine 2000 (Invitrogen). Cells were lysed for protein extraction 24 h after transfection using a standard lysis buffer (150 mM NaCl, 50 mM Tris(hydroxymethyl)aminomethane (TRIS) pH 7.4, 5 mM ethylenediaminetetraacetic acid (EDTA), 1% Triton x-100, 1 mM phenylmethylsulfonyl fluoride (PMSF), and protease inhibitor cocktail). Cells were lifted using a cell scraper and then passed through a 22.5-gauge needle before transferring lysates to 1.5 ml microfuge tubes. Lysates were then transferred to a rotator at 4^°^C for 30 min. After 30 min, lysates were centrifuged at 12,000 rpm for 10 min at 4^°^C. Supernatant was removed and flash frozen in liquid nitrogen for long term storage. In preparation for immunoblotting, protein samples were thawed on ice. For all blots, 10 μg protein per sample was denatured at 95^°^C and loaded onto freshly poured 8% sodium dodecyl sulfate–polyacrylamide gel electrophoresis (SDS-PAGE). Gels were run for 90 min at 120 V and transferred to nitrocellulose membrane for 1 h at 100 V. Membranes were blocked in 5% milk diluted in tris-buffered saline with tween-20 (TBST) for 1 h. For testing the expression of AMPAR chimeras, membranes were incubated with both rat anti-HA antibodies and mouse anti-tubulin antibodies at 4^°^C overnight. For testing the expression of SD4/IFITM3 chimeras, membranes were incubated with anti-SD4, anti-IFITM3, and anti-tubulin antibodies at 4^°^C overnight. Membranes were washed with TBST and incubated in HRP conjugated goat anti-rat and HRP-conjugated goat anti-mouse secondary antibodies for 1 h at room temperature. Luminata Crescendo reagent was added to membrane for the direct detection of HRP signal (Azure Biosystems).

### Immunocytochemistry

#### COS Cells

COS cells were plated in 6-well plates containing coverslips coated with poly-L-lysine (Sigma-Aldrich). Cells were plated at a density of 300,000 cells per well and cultured for 24 h prior to transfection. All transient transfection experiments contained a total of 2 μg of DNA (1.75 μg receptor and 250 ng of either SD4 or pRK5 empty vector) using Lipofectamine 2000 (Invitrogen) and cells were cultured for an additional 24 h. For live labeling, cells were first incubated at 4^°^C for 10 min. Cells were washed once with cold PBS and incubated in rat anti-HA antibody diluted in COS media for 20 min at 4^°^C. After primary staining, cells were washed three times with cold PBS and incubated in donkey Alexa 594-conjugated anti-rat secondary antibody diluted in COS media for 20 min. Cells were washed three times with cold PBS and then with warm COS media. Plates were transferred back to 37^°^C incubator for 30 min. Cells were washed with PBS and fixed in 4% paraformaldehyde (PFA) for 10 min.

For staining of total SD1 or total SD4, coverslips were incubated in 0.1% Triton-X100 diluted in PBS for 15 min. Cells were blocked with 5% milk in PBS for 30 min and incubated in primary antibody for 1.5 h at room temperature. Coverslips were washed three times with PBS and incubated in donkey Alexa 488-conjugated anti-mouse IgG2a for 1 h. Coverslips were washed three times with PBS and mounted on slides with Fluoromount G (Southern Biotech).

#### Primary Neurons

For chemical long-term potentiation (LTP), hippocampal neurons at DIV 12–14 were equilibrated in artificial cerebrospinal fluid (aCSF) containing 2 mM magnesium (Mg^+2^) and 2 mM calcium (Ca^+2^) at 37^°^C in incubator for 30 min. Neurons were washed with PBS and replaced with aCSF containing the treatment buffer (2 mM Ca^+2^; 200 μM glycine; 20 μM bicuculine; 3 μM strychnine), or a vehicle control. Strychnine was diluted in DMSO while glycine and bicuculine were diluted in water, so an equivalent amount of dimethyl sulfoxide (DMSO) or water, respectively, was added as the vehicle control. Neurons were incubated at 37^°^C for 5 min for chemical-LTP induction. Coverslips were then transferred to a recovery buffer (aCSF w/Mg^+2^; no drugs) for 20 min at 37^°^C. For labeling of synaptic GluA1, neurons were washed 3 times with PBS and incubated with anti-GluA1 antibody against the extracellular N-terminus diluted in PBS with 3% bovine serum albumin (BSA) at 37^°^C for 1 h. Cells were then washed with PBS, fixed with 4% PFA, and then permeabilized with 0.1% Triton X-100 for 15 min. Neurons were blocked with 10% BSA for 30 min. Neurons were then stained for total anti-SD4 and total anti-vGlut1 overnight in 3% BSA at 4^°^C. After incubation, coverslips were washed 3 times with PBS and incubated in secondary antibodies for each marker for 1 h at room temperature. Neurons were then washed 3 times with PBS and mounted on glass microscope slides for imaging.

### Image Analysis

For quantitative analyses, images were taken using either an Olympus FluoView 1000 or Zeiss LSM510 confocal microscope with a 63 × /1.5 NA oil objective with identical settings for laser power, photomultiplier gain, and digital offset. Pinhole (1 AU) and resolution (1,024 × 1,024 pixels) were constant for all images.

Images were analyzed blinded to the experimental condition. Image files were imported into image analysis software (ImageJ) to determine the average size of clusters for each condition. Selected cells were cropped from the original images, saved blinded and subjected to the analysis by an individual not involved in the cell selection and blinding process. The threshold for each independent experiment is determined by averaging the thresholds of at least 25% of images within a dataset. Threshold values were determined by duplicating each image and adjusting the threshold of the duplicated image converted to black and white. Thresholds were determined such that all recognizable puncta were included in the analysis. The average threshold was then applied to all images within a dataset for cluster analysis by inserting values into a pre-written script run through the ImageJ software. The script separates the channels, applies the average threshold values, creates the mask overlay, and analyzes cluster parameters (number and size) defined by the mask. Clusters within the range of 0.1–3.5 μm^2^ were measured. After data collection and the unblinding process, the puncta size of all signals was subjected to statistical analysis. For analysis of puncta size based on co-localization with SD4 (stratification analysis), co-localization was defined as overlap of ≥ 1 pixel. A mask overlay was then created using ImageJ by overlapping the two channels of sGluA1 and SD4. The colocalized puncta in the image representing the receptor coexpressed with SD4 were then used to select unambiguous single puncta manually in the receptor mask overlay. XY coordinates were used to confirm the selected puncta in the receptor mask overlay that corresponded with the colocalized puncta in the image for the receptor coexpressed with SD4. For figure preparation, signals were adjusted for all panels within a figure by using the equal linear adjustments of levels in Photoshop (Adobe Systems).

### Statistical Analysis

Data were collected from at least two independent experiments and a minimum *n* = 10–15 cells per condition per experiment. All graphs and statistical analyses were generated using GraphPad Prism software. Graphs depict the data average and the standard error of the mean (SEM). Statistical significance was assessed by one-way ANOVA with *post-hoc* Tukey’s test or Student’s *t*-test. Significance is defined as *p < 0.05; ^**^p < 0.01; ^***^p < 0.001; and ^****^p < 0.0001.

## Results

### SD4 Clusters GluA1 and GluA2 Containing AMPA Receptors

To characterize the relationship between AMPARs and SD4, we used a clustering assay previously established within our lab (Kalashnikova et al., 2010). The full-length AMPAR subunits GluA1 and GluA2, as well as the kainate receptor subunit (GluK2), were expressed in heterologous COS cells either alone or co-expressed with full-length HA-tagged SD4. GluK2 is predicted not to associate with SD4 and served as a negative control. Each receptor subunit contains an N-terminal HA tag for extracellular detection. COS cells were first live-labeled with anti-HA antibodies to examine the distribution of surface expressing GluA1, GluA2, or GluK2. The receptors have extracellular N-termini, while SD4 does not, so only surface GluA1, GluA2, or GluK2 were labeled. After fixation and permeabilization, cells were stained with anti-SD4 antibodies for total SD4. It is important to note that SD4 is a type II transmembrane protein while GluA1 and GluA2 are type I transmembrane proteins; therefore, the HA epitope for the live-labeling of GluA1 or GluA2 is not accessible to HA-SD4 even when the anti-HA antibody is internalized because the HA-tag on SD4 faces the cytoplasm while the anti-HA antibody remains lumenal. Furthermore, the anti-HA antibody used for the surface labeling of HA-GluA1 or HA-GluA2 does not label HA-SD4 when expressed alone (as shown below).

**FIGURE 1:**
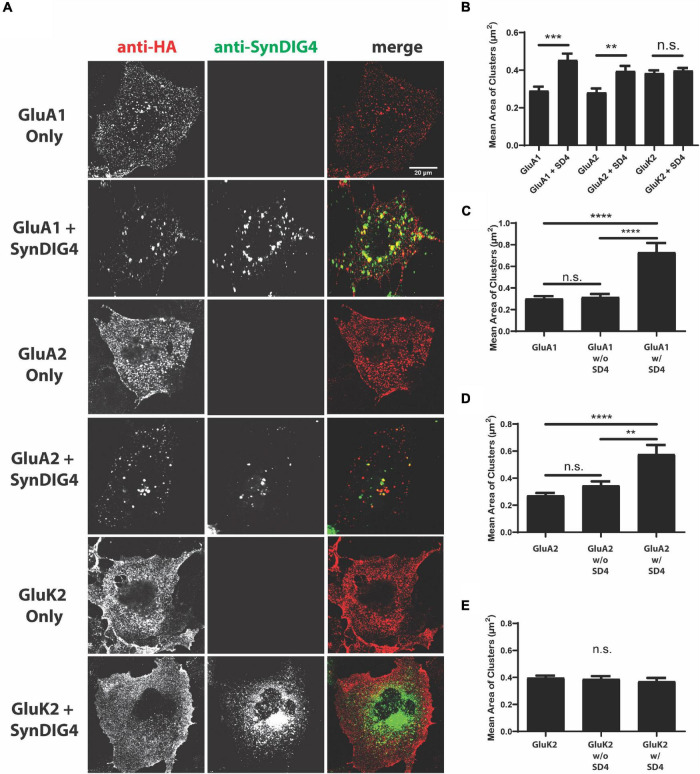
Synapse differentiation-induced gene 4 (SD4) clusters GluA1 and GluA2-containing α-amino-3-hydroxy-5-methyl-4-isoxazolepropionic acid receptors (AMPARs). **(A)** Representative confocal images of COS cells transfected with either receptor alone, or co-transfected with both receptor and SD4. Cells were live labeled with anti-hemagglutinin (HA) antibodies against surface expressing receptors and anti-SD4 antibodies for total SD4. Scale bar = 20 μm. **(B)** Graph depicts the mean cluster size of GluA1 alone (*n* = 10), GluA1 + SD4 (*n* = 12), GluA2 alone (*n* = 16), GluA2 + SD4 (*n* = 10), GluK2 alone (*n* = 13), or GluK2 + SD4 (*n* = 11) puncta. **(C–E)** Graphs depict the stratification of GluA1 **(C)**, GluA2 **(D)**, or GluK2 **(E)** puncta either co-localized or not co-localized with SD4 compared with the average cluster size of receptor alone. Data are represented as mean cluster size ± SEM; n.s. not significant; **p < 0.01; ***p < 0.001; and ****p < 0.0001; one-way ANOVA with *post-hoc* Tukey’s test.

We observed diffuse and even distribution of GluA1, GluA2, and GluK2 when expressed alone. When co-expressed with SD4, a change in the overall distribution of both GluA1 and GluA2 was observed (Figure 1). No difference was observed when GluK2 was co-expressed with SD4, indicating the specificity of SD4 for AMPARs (Figure 1A). Quantification indicates a significant increase in the mean cluster size of GluA1 and GluA2 puncta when co-expressed with SD4 compared with receptor alone (Figure 1B). Although GluK2 puncta are larger at baseline, there is no significant change in puncta size when co-expressed with SD4 (Figure 1B).

We noted a distribution of GluA1 or GluA2 cluster sizes in SD4 co-expressing cells. To determine whether cluster size was related to overlap with SD4, which was not captured in the previous analysis, we stratified populations in co-expressing cells into two groups representing glutamate receptor puncta co-localized with SD4 (w/SD4) or not co-localized with SD4 (w/o SD4). The stratification of GluA1 puncta co-localized with SD4 showed that the mean cluster size is significantly greater when SD4 and GluA1 are co-localized compared to GluA1 expressed alone (Figure 1C). In contrast, the size of puncta not co-localized with SD4 were not significantly different compared with GluA1 alone (Figure 1C). In addition, the mean size of GluA2 clusters is significantly greater when co-localized with SD4, while non-colocalized clusters are not significantly different compared with GluA2 alone (Figure 1D). No significant differences were observed in the size of GluK2 clusters co-localized with SD4 or not co-localized compared with GluK2 alone (Figure 1E). These results provide evidence that the co-expression of SD4 is sufficient to re-distribute and cluster GluA1 and GluA2-containing AMPARs in heterologous cells, and the increased cluster size is dependent on co-localization with SD4.

### The Proline-Rich N-Terminus of SD4 Is Dispensable for Clustering With GluA1 and GluA2

**FIGURE 2:**
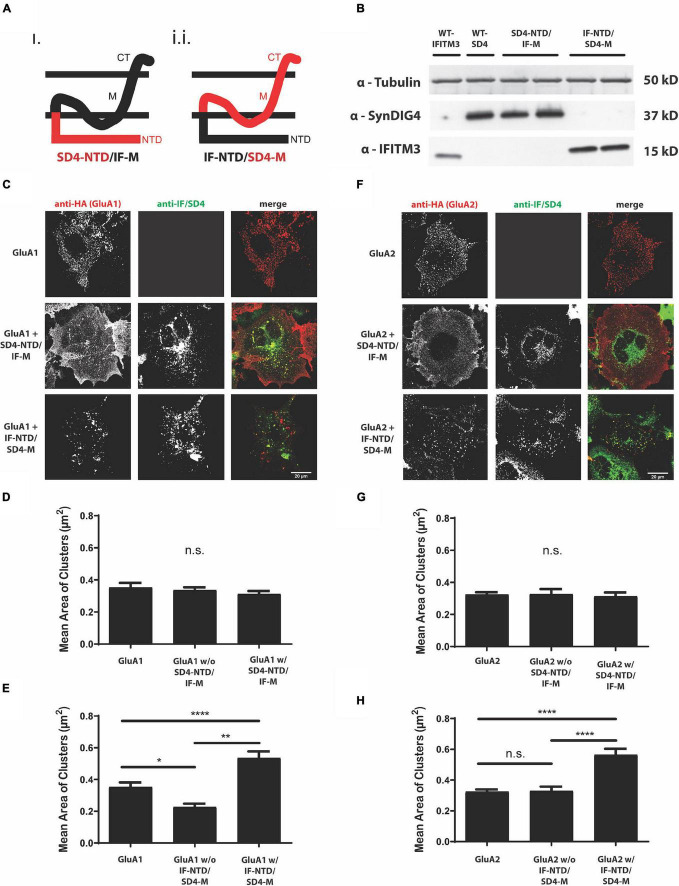
The proline rich N-terminus of SD4 is dispensable for clustering with GluA1 and GluA2. **(A)** Schematic depicting the chimeric protein structures. Chimeras were generated expressing either (i) the N-terminus of SD4 and membrane domain of IFITM3 (SD4-NTD/IF-M) or (ii) the N-terminus of IFITM3 and membrane domain of SD4 (IF-NTD/SD4-M) (as shown in Table 1 of Methods). **(B)** Immunoblot of COS cell lysates transfected with SD4 and IFITM3 chimeras. β-tubulin was used as the loading control. **(C)** Representative confocal images depict either GluA1 expressed alone or co-expressed with either IF/SD4 chimera. Scale bar = 20 μm. **(D,E)** Graph depicts the mean cluster size of GluA1 puncta from the stratification of GluA1 co-localized or not co-localized with SD4-NTD/IF-M chimeras **(D)** or IF-NTD/SD4-M chimeras **(E)** compared with GluA1 alone. GluA1 alone (*n* = 10), GluA1 + SD4-NTD/IF-M (*n* = 12), and GluA1 + IF-NTD/SD4-M (*n* = 12). **(F)** Representative confocal images of GluA2 alone or co-expressed with IF/SD4 chimeras. Scale bar = 20 μm. **(G,H)** Graph depicts the mean cluster size of GluA2 puncta from the stratification of GluA2 co-localized or not co-localized with SD4-NTD/IF-M chimeras **(G)** or IF-NTD/SD4-M chimeras **(H)** compared with GluA2 alone. GluA2 alone (*n* = 13), GluA2 + SD4-NTD/IF-M (*n* = 12), and GluA2 + IF-NTD/SD4-M (*n* = 16). Data are represented as mean cluster size ± SEM; n.s. not significant; *p < 0.05; **p < 0.01; ****p < 0.0001; one-way ANOVA with *post-hoc* Tukey’s test.

To identify the region of SD4 sufficient for clustering with GluA1 or GluA2, we generated chimeric proteins by swapping domains between SD4 and the distantly related Dispanin family (Sällman Almén et al., 2012) member IFITM3 with a similar topology (Yount et al., 2010; Ling et al., 2016). One chimera was generated using the intracellular N-terminal region of SD4 and the C-terminal domains of IFITM3, such as the hydrophobic segment that does not span the lipid bilayer, the small intracellular loop, the transmembrane domain, and the short extracellular portion (SD4-NTD/IF-M), and a second chimera was generated using the intracellular N-terminal region of IFITM3 and the corresponding C-terminal domains of SD4 (IF-NTD/SD4-M) (Figure 2A and Table 1). For brevity, we refer to the C-terminal domains as the “membrane associated region.” Constructs were first verified by immunoblot with antibodies that only recognize the N-terminus of their respective proteins (Figure 2B). Therefore, signal is only present when the N-terminus is expressed.

Furthermore, GluA1 was expressed in COS cells either alone, or co-expressed with full-length SD4, SD4-NTD/IF-M, or IF-NTD/SD4-M (Figure 2C). We observed no difference in the mean cluster size of GluA1 populations either co-localized or not co-localized with SD4-NTD/IF-M compared with GluA1 alone (Figure 2D). However, the stratification of GluA1 populations indicated a significant increase in the mean size of GluA1 clusters when co-localized with IF-NTD/SD4-M compared with GluA1 alone (Figure 2E). Next, GluA2 was expressed in COS cells either alone, or co-expressed with either IF/SD4 chimeras (Figure 2F). The stratification of GluA2 populations co-localized or not co-localized with SD4-NTD/IF-M resulted in no change in the mean size of GluA2 clusters (Figure 2G). However, we observed a significant increase in the mean size of GluA2 clusters when co-localized with IF-NTD/SD4-TM compared with GluA2 alone (Figure 2H).

These results indicate that the intracellular proline-rich N-terminal portion of SD4 is dispensable for clustering with GluA1 and GluA2. Furthermore, the C-terminal portion of SD4, which consists primarily of membrane associated regions, is sufficient for clustering with GluA1- and GluA2-containing AMPARs, and that clustering of AMPARs is dependent on co-localization with SD4.

### The N-Terminus of GluA1 Is Dispensable for Clustering With SD4

**FIGURE 3:**
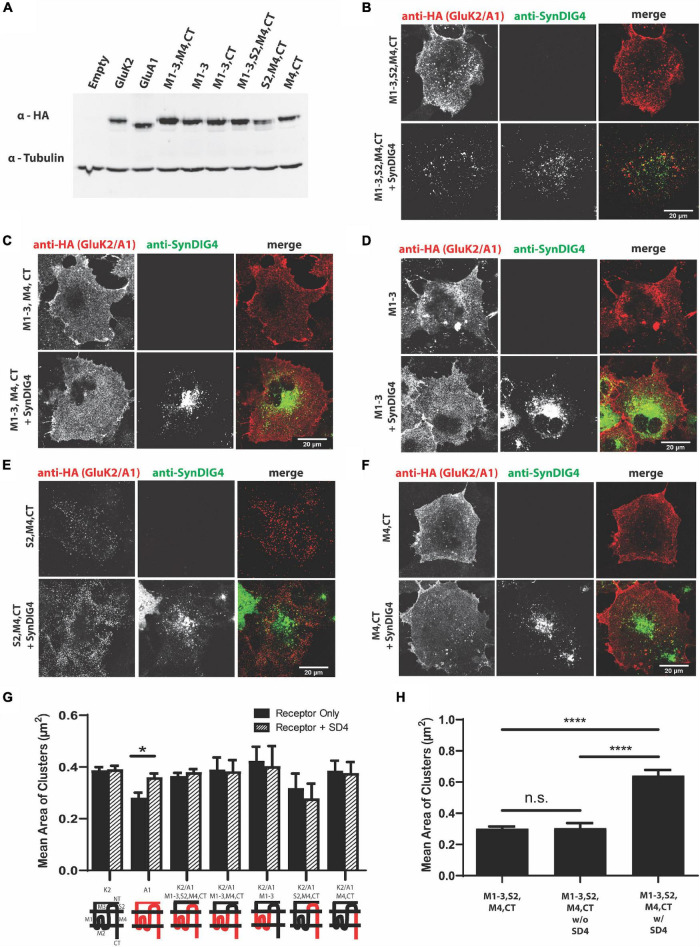
The N-terminus of GluA1 is dispensable for clustering with SD4. **(A)** Immunoblot depicting expression of GluK2/GluA1 chimeras. Homologous domains of GluA1 were inserted into the backbone of GluK2 (as shown in Table 2 of Methods). β-tubulin was used as the loading control. **(B–F)** Representative confocal images of COS cells transfected with either a GluK2/A1 chimeric receptor alone, or co-transfected with SD4. Scale bar = 20 μm. **(G)** Graph depicts the mean area of clusters when GluK2, GluA1, and each GluK2/A1 chimera is expressed either alone or co-expressed with SD4. **(H)** Graph depicts the mean cluster size of M1-3, S2, M4, CT chimera stratified for either co-localized or not co-localized with SD4. M1-3, S2, M4 alone (*n* = 11), M1-3, S2, M4 w/o SD4 (*n* = 13), M1-3, S2, M4 w/SD4 (*n* = 13). Data are represented as mean ± SEM; n.s. not significant; *p < 0.05; ****p < 0.0001; one-way ANOVA with *post-hoc* Tukey’s test.

To identify the region of GluA1 that is sufficient for clustering with SD4, we generated chimeric GluA1 proteins using the homologous domains of GluK2 (Table 2). The expressions of all GluK2/GluA1 chimeras were verified by immunoblot (Figure 3A). The chimeras were then transfected and expressed in COS cells either alone or with full-length SD4 (Figures 3B–F). The quantification of mean area of clusters shows an increase in puncta size when SD4 is co-expressed with GluA1, but no significant difference when co-expressed with the chimera expressing the M1-3, S2, M4, and CT domains of GluA1 (Figure 3G). However, the stratification of GluK2/A1/M1-3/S2/M4/CT chimeric puncta (Figure 3B) depicts a significant increase in puncta size when co-localized with SD4 (Figure 3H). Therefore, we conclude that the N-terminus of GluA1 is dispensable for clustering with SD4, and cluster size is dependent on co-localization with SD4.

### The N-Terminus of GluA2 Is Dispensable for Clustering With SD4

**FIGURE 4:**
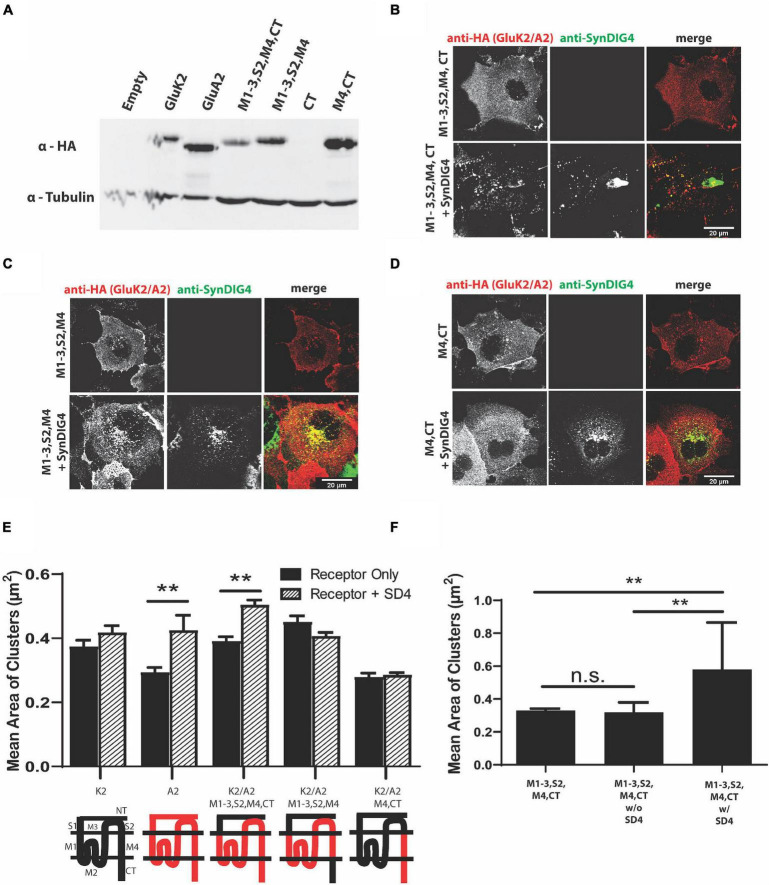
The N-terminus of GluA2 is dispensable for clustering with SD4. **(A)** Immunoblot depicting expression of GluK2/GluA2 chimeras. Homologous domains of GluA2 were inserted into the backbone of GluK2 (as shown in Table 3 of Methods). β-tubulin was used as the loading control. **(B–D)** Representative confocal images of COS cells transfected with either a GluK2/A2 chimeric receptor alone, or co-transfected with SD4. Scale bar = 20 μm. **(E)** Graph depicts the mean area of clusters when GluK2, GluA2, and each GluK2/A2 chimera is expressed either alone or co-expressed with SD4. **(F)** Graph depicts mean the area of M1-3, S2, M4, CT chimera clusters stratified for either co-localized or non-colocalized with SD4. M1-3, S2, M4, CT alone (*n* = 14), M1-3, S2, M4, CT w/o SD4 (*n* = 11), M1-3, S2, M4, CT w/SD4 (*n* = 13). Data are represented as mean ± SEM; n.s. not significant; **p < 0.01; one-way ANOVA with *post-hoc* Tukey’s test.

We next generated GluA2 chimeras using the homologous domains of GluK2 (Table 3). An expression of GluA2 chimeras was verified by immunoblot (Figure 4A). All chimeras were transfected in COS cells either alone or with full-length SD4 (Figures 4B–D). We found that only the chimera expressing the M1-3, S2, M4, and CT domains of GluA2 resulted in an altered distribution when co-expressed with SD4 (Figure 4B). Additionally, the GluK2/A2/M1-3/S2/M4 chimera, where the GluA2-CT domain was not present, resulted in a loss of the clustering phenotype (Figure 4C). Therefore, these experiments show the importance of GluA2 C-terminal domain for clustering with SD4. The quantification of mean area of clusters shows a significant increase in cluster size when SD4 is co-expressed with either full-length GluA2 or the GluK2/A2/M1-3/S2/M4/CT chimera (Figure 4E). Furthermore, the stratification of puncta from Figure 4B shows a significant increase in puncta area only when co-localized with SD4 (Figure 4F). Therefore, we conclude that the N-terminus of GluA2 is dispensable for clustering with SD4, and cluster size is dependent on co-localization with SD4.

### The M4 and C-Terminus of GluA2 Is Sufficient for Clustering With SD1

**FIGURE 5:**
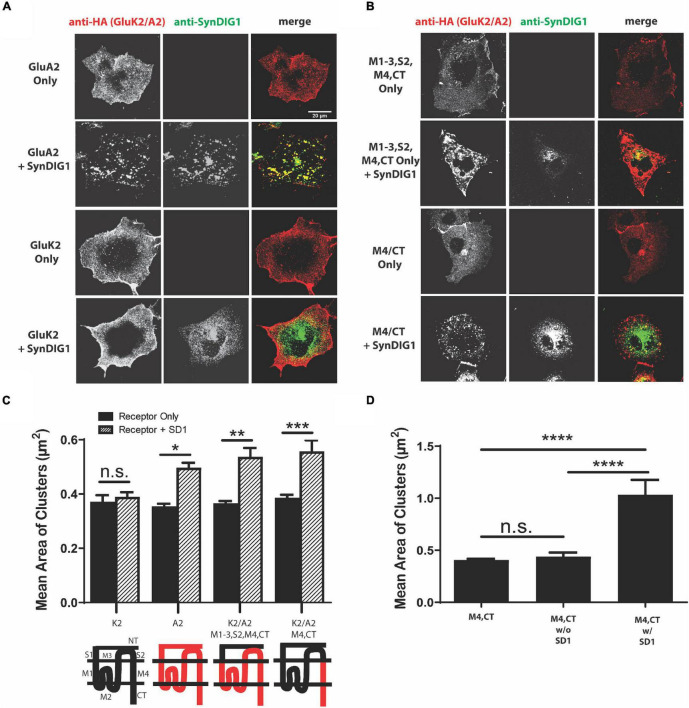
The M4 and C-terminus of GluA2 is sufficient for clustering with synapse differentiation-induced Gene 1 (SD1). **(A)** Representative confocal images of COS cells transfected with GluA2 alone, GluK2 alone, or co-transfected with GluA2 or GluK2 and SD1. Scale bar = 20 μm. **(B)** Representative confocal images of chimeras expressing the M1-3, S2, M4, CT and M4, CT domains of GluA2 either alone or co-expressed with SD1. Scale bar = 20 μm. **(C)** Graph depicts the mean area of clusters when GluK2, GluA2, and each GluK2/A2 chimera is expressed either alone or co-expressed with SD1. **(D)** Graph depicts the mean area of M4, CT chimera clusters stratified for either co-localized or non-colocalized with SD1. M4, CT alone (*n* = 13), M4, CT w/o SD1 (*n* = 13), M4, CT w/SD1 (*n* = 11). Data are represented as mean ± SEM; n.s. not significant; *p < 0.05; **p < 0.01; ***p < 0.001; ****p < 0.0001; one-way ANOVA with *post-hoc* Tukey’s test.

For comparison, we sought to identify a region of AMPAR necessary for clustering with the SD4-related family member SD1. Full-length GluA2 had previously been observed to cluster with SD1 (Kalashnikova et al., 2010); therefore, GluA2 and GluK2 were used as positive and negative controls, respectively. We observed altered distribution of GluA2, but not GluK2, when co-expressed with SD1 (Figure 5A). Next, we co-expressed two of the key GluA2 chimeras from Figure 4. Similar to SD4, we observed clustering when the GluK2/A2/M1-3/S2/M4/CT chimera was co-expressed with SD1. However, in contrast to SD4, we observed clustering when the GluK2/A2/M4/CT chimera was co-expressed with SD1 (Figure 5B). Quantification depicts a significant increase in the mean area of clusters when SD1 is co-expressed with GluK2/A2/M1-3/S2/M4/CT and GluK2/A2/M4/CT, but not with GluK2 (Figure 5C). Last, the stratification of M4 and CT chimera puncta co-localized with SD1 results in a significant increase in the mean area of clusters (Figure 5D). Therefore, the M4 and CT of GluA2 is sufficient for clustering with SD1.

### SD4 Cluster Size Increases When Colocalized With GluA1 and GluA2

**FIGURE 6:**
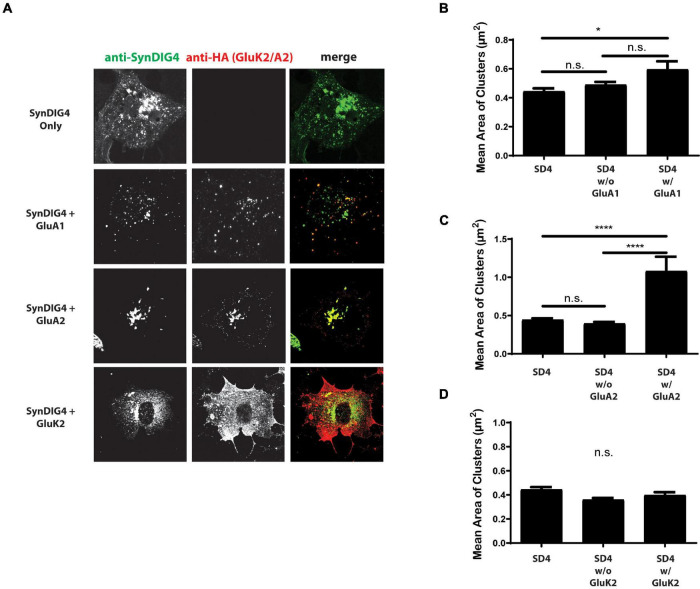
Synapse differentiation-induced gene 4 cluster size increases when colocalized with GluA2. **(A)** Representative confocal images of COS cells expressing SD4 alone, or SD4 co-expressed with either GluA1, GluA2 or GluK2. Scale bar = 20 μm. **(B–D)** Graph depicts the mean area of SD4 clusters stratified for either co-localized or non-colocalized with GluA1 **(B)**, GluA2 **(C)**, or GluK2 **(D)**. **(B)** SD4 alone (*n* = 12), SD4 w/o A1 (*n* = 10), SD4 w/A1 (*n* = 10). **(C)** SD4 alone (*n* = 12), SD4 w/o A2 (*n* = 10), SD4 w/A2 (*n* = 10). Data are represented as mean ± SEM; n.s. not significant; *p < 0.05; ****p < 0.0001; one-way ANOVA with *post-hoc* Tukey’s test.

Next, we were interested in whether the co-expression of AMPARs with SD4 results in a reciprocal increase in the cluster size of SD4. For these experiments, SD4 was expressed in COS cells either alone, or co-expressed with either GluA1, GluA2, or GluK2 (Figure 6A). The stratification of SD4 puncta co-localized with GluA1 results in a significant increase in the mean cluster size of SD4 puncta co-localized with GluA1 compared with SD4 alone (Figure 6B). Additionally, the mean cluster size of SD4 puncta co-localized with GluA2 is also significantly increased (Figure 6C). We observed no difference in the mean cluster size of SD4 puncta whether co-localized or not-colocalized with GluK2 compared with SD4 alone (Figure 6D). We conclude that not only does the co-localization of SD4 with AMPARs increase the mean cluster size of the receptor, but colocalization with AMPARs also significantly increase the cluster size of SD4.

### SD4 Clustering of GluA1 and GluA2 Is Temperature Dependent

**FIGURE 7:**
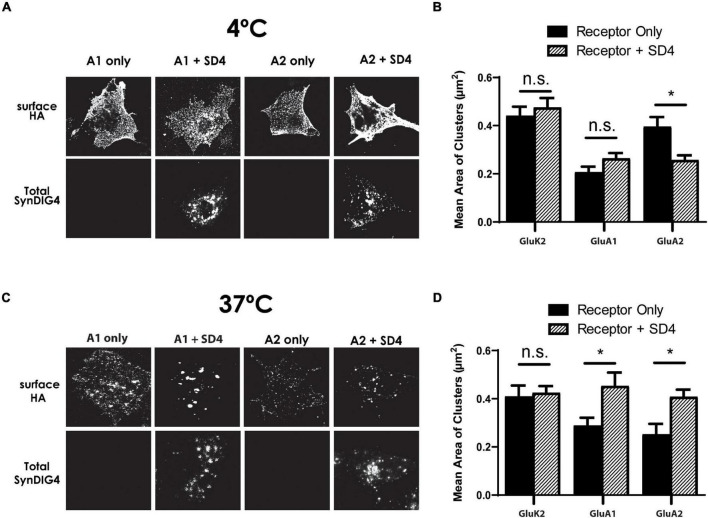
Synapse differentiation-induced gene 4 clustering of GluA1 and GluA2 requires incubation at 37^°^C. **(A)** Representative confocal images of COS cells expressing either GluK2, GluA1, or GluA2 lone or co-expressed with SD4 after incubation at 4^°^C. **(B)** Graph depicts the mean area of receptor clusters. GluK2 alone (*n* = 14), GluK2 + SD4 (*n* = 12), GluA1 alone (*n* = 10), GluA1 + SD4 (*n* = 14), GluA2 alone (*n* = 12), GluA2 + SD4 (*n* = 13). **(C)** Representative confocal images of COS cells expressing either GluK2, GluA1, or GluA2 alone or co-expressed with SD4 after incubation at 37^°^C. GluK2 alone (*n* = 11), GluK2 + SD4 (*n* = 16), GluA1 alone (*n* = 10), GluA1 + SD4 (*n* = 10), GluA2 alone (*n* = 10), and GluA2 + SD4 (*n* = 10). **(D)** Graph depicts mean area of receptor clusters. Data are represented as mean ± SEM; n.s. not significant; *p < 0.05; one-way ANOVA with *post-hoc* Tukey’s test.

A clustering of GluA2 by SD1 requires a 37^°^C incubation after the surface labeling at 4^°^C (Kalashnikova et al., 2010). This observation suggests that a biological process, such as endocytosis is necessary for clustering by SD1. Then, we were interested to determine whether SD4 clustering of AMPARs is temperature dependent. For these experiments, duplicate plates of COS cells expressing GluK2, GluA1, or GluA1 either alone or co-expressed with SD4 were prepared. For the surface labeling of receptors, all plates were incubated at 4^°^C. Next, one plate was transferred to a 37^°^C incubator, while the other plate remained at 4^°^C. After incubation, coverslips were fixed and imaged. We observed that incubation at 4^°^C does not result in a change of distribution for any of the receptors, either alone or co-expressed with SD4 (Figure 7A). Quantification indicates no significant increase in the mean area of clusters after the 4^°^C incubation (Figure 7B). However, incubation at 37^°^C resulted in the clustering of receptors as expected (Figure 7C). Quantification shows a significant increase in the mean area of clusters when either GluA1 or GluA2 are co-expressed with SD4, but not GluK2 (Figure 7D). Therefore, we conclude that the endocytosis of surface labeled AMPARs is most likely to be necessary for clustering with SD4.

### Co-localization of SD4 With Synaptic GluA1 Is Increased After Chemical-Long-Term Potentiation

**FIGURE 8:**
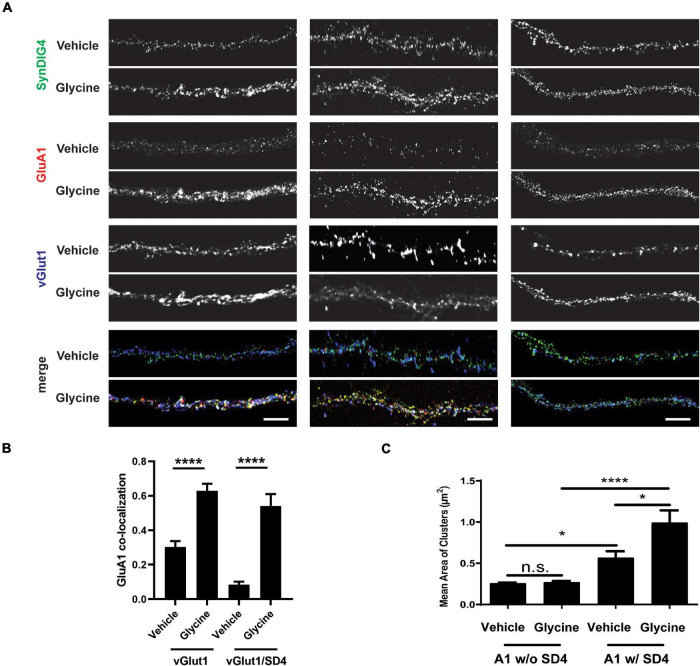
Co-localization of SD4 with synaptic GluA1 is increased after chemical-LTP. **(A)** Representative confocal images of dendritic stretches from primary rat hippocampal neurons at DIV13. Three dendritic stretches are shown for each condition. Neurons were treated with either vehicle or 200 μM glycine in artificial cerebrospinal fluid (aCSF) without magnesium at 37^°^C for 5 min. Neurons were transferred to aCSF recovery buffer (without drugs) for 20 min and then stained with anti-GluA1 antibody at 37^°^C for 1 h. Neurons were then fixed, permeabilized, and stained for total SD4 and vGlut1. **(B)** Quantification of co-localization between GluA1, SD4, and vGlut1 upon incubation with vehicle or glycine. **(C)** Quantification of mean area of GluA1 clusters either co-localized or non-colocalized with SD4 after treatment with vehicle or glycine. Data represented as mean ± SEM; n.s., non-significant; *n* = 15 cells per condition; *p < 0.05; ****p < 0.0001; Student’s *t*-test **(B)**; one-way ANOVA with *post-hoc* Tukey’s test. **(C)** Scale bar = 5 μm.

To test the role of SD4-dependent AMPAR clustering in synaptic plasticity, we utilized the primary culture of dissociated rat hippocampal neurons. Neurons were treated with 200 μM glycine or vehicle in aCSF without magnesium at 37^°^C for 5 min to induce chemical-LTP. Neurons were then transferred to aCSF recovery buffer for 20 min and then stained with anti-GluA1 antibody at 37^°^C for 1 h. Neurons were then fixed, permeabilized, and stained for total SD4 and vGlut1 (Figure 8A; three representative stretches from three individual neurons are shown). The percentage of synaptic GluA1 puncta (defined as overlap with the pre-synaptic marker vGlut1) increased by 2-fold after glycine treatment compared with vehicle (Figure 8B), indicating successful chemical-LTP induction. Additionally, the percentage of synaptic GluA1 puncta co-localized with SD4 significantly increased after chemical-LTP (Figure 8B), suggesting that at least a portion of SD4 redistributes to the synapse during glycine induced chemical-LTP. The average number of vGlut1 puncta per stretch was equivalent (vehicle: 27.02 ± 2.39, *n* = 42; glycine: 25.27 ± 1.73, *n* = 45; *p* = 0.553), indicating that the increased percentage of synaptic GluA1 which colocalizes with SD4 is not a result of changes in vGlut1.

Next, we looked at changes in the mean area of GluA1 clusters as a result of chemical-LTP. We stratified these data to determine whether changes in the size of these clusters are dependent on co-localization with SD4. We observed that there was no change in the mean area of GluA1 clusters not colocalized with SD4 after treatment with glycine compared with vehicle (Figure 8C). Interestingly, there was a significant increase in the size of GluA1 clusters during vehicle treatment only when GluA1 was co-localized with SD4. Furthermore, we observed a significant increase in the mean area of GluA1 clusters co-localized with SD4 after glycine induced chemical-LTP (Figure 8C). As a result of these experiments, we conclude that the co-localization of SD4 with synaptic GluA1 increases after chemical-LTP, and this co-localization results in an increase in the mean area of GluA1 clusters.

## Discussion

Previously, we showed that SD4 alters AMPAR biophysical properties in a subunit-specific manner (Matt et al., 2018), indicating a direct and specific interaction with AMPARs. Indeed, SD4 has been identified in multiple independent proteomic studies as a component of AMPAR complexes (von Engelhardt et al., 2010; Schwenk et al., 2012; Shanks et al., 2012) as well as recent structural studies of native AMPAR complexes from brain (Yu et al., 2021). Although present in synaptosomal membranes, SD4 is de-enriched in the PSD where the majority of SD4 overlaps with GluA1 outside of synapses (Kirk et al., 2016), suggesting that SD4 associates primarily with extra-synaptic AMPARs. Here we present evidence that SD4 and GluA1 or GluA2 AMPARs bi-directionally increase the cluster size of each other in heterologous cells. Distinct regions within SD4, GluA1 and GluA2 are critical for this mutually dependent clustering activity. Intriguingly, the bi-directional clustering requires incubation at 37^°^C, suggesting that an endocytosis of surface labeled AMPARs is most likely necessary for clustering with SD4. Indeed, a recent study demonstrated overlap of SD4 with early endosomes in hippocampal neurons (Martin et al., 2021), consistent with its role in clustering AMPARs that have been internalized. Importantly, the increased cluster size of GluA1 that overlaps with SD4 is observed in primary hippocampal neurons upon chemical-LTP, suggesting that this clustering activity is a mechanism underlying the strengthening of synapses during synaptic plasticity. Our current model is that the SD4-induced clustering of AMPARs occurs intracellularly after endocytosis to establish a reserve pool of intracellular extrasynaptic AMPARs that can be deployed to the cell surface during LTP. These intracellular clusters of AMPARs co-localized with SD4 presumably remain intracellular in heterologous cells. In addition, others have shown that SD4 KO mice are deficient for LTD (Troyano-Rodriguez et al., 2019); thus, the intracellular clustering of AMPARs could also be employed during LTD as a mechanism to restrict the surface accumulation of AMPARs perhaps upon differential regulation. Additional experiments beyond the scope of this study are needed to investigate the effects of SD4 on AMPAR trafficking in neurons during synaptic plasticity.

Synapse differentiation-induced gene 4 is predicted to contain two membrane-associated domains, with only one that spans the membrane, a large proline-rich intracellular N-terminus, a small intracellular loop, and a small extracellular C-terminus (Kirk et al., 2016), confirmed in a recent study (Martin et al., 2021). Proline residues have often been linked to protein–protein interactions (Kay et al., 2000; Freund et al., 2008). However, our results indicate that only the C-terminal region, such as the membrane bound domains, intracellular loop, and small extracellular tail of SD4 is important for clustering with GluA1 or GluA2. Others reported that SD4 is able to co-immunoprecipitate a small amount (2% of input) of GluA1 or GluA2 when coexpressed in HEK293 cells (Martin et al., 2021). Furthermore, deletion of the intracellular loop, the transmembrane domain, and the small extracellular tail of SD4 eliminated the observed co-immunoprecipitation (Martin et al., 2021), consistent with our results indicating that the proline-rich intracellular N-terminal region is not required for clustering. SD4 does not contain a PDZ binding motif and it is not enriched in the PSD (Kirk et al., 2016). The proline-rich domain may be important for interaction with other auxiliary factors or scaffolds necessary for trafficking and anchoring at synapses. Additional experiments will address this possibility.

To identify the GluA1 AMPAR domain sufficient for clustering with SD4, we used GluK2/GluA1 chimeras which swap homologous protein domains between receptors. All GluK2/GluA1 chimeras lacked the NT domain of GluA1. The total mean area of GluA1 puncta was not significantly larger compared with the chimeras when co-expressed with SD4. However, we did observe some clustering with the GluK2/A1/M1-3/S2/M4/CT chimera when expressed alone, which may have occluded any increase in cluster size in this analysis. In support of this possibility, the mean cluster size of GluK2/A1/M1-3/S2/M4/CT chimeric puncta is significantly increased by the stratification of puncta co-localized with SD4, while no increase is observed by the stratification of other chimeras co-localized with SD4. We observed altered distribution of the receptor only when the entire membrane, S2, and CT domains (GluK2/A1/M1-3/S2/M4/CT) were present and co-localized with SD4 in COS cells. Therefore, we conclude that the NT domain is dispensable, while the entire membrane bound domain of GluA1, in addition to the S2 and CT domains, are necessary for clustering by SD4.

Similar to the GluA1 chimeras, we used GluK2/GluA2 chimeras to identify the region sufficient for clustering with SD4. All GluK2/GluA2 chimeras lacked the NT domains of GluA2. We observed a change in distribution when the entire membrane bound domain, S2 domain, and CT domain of GluA2 (GluK2/A2/M1-3/S2/M4/CT) was expressed with SD4. In these experiments, we saw a significant increase in mean puncta size, with no significant differences with any additional chimeras. Interestingly, we observed that clustering was lost when the CT domain was absent, indicating an importance of the CT for clustering with SD4. Additionally, we did not observe clustering when the M1-3 and S2 domains were absent. We conclude that the entire membrane bound domain of GluA2, in addition to the S2 and CT domains, is necessary for clustering by SD4.

Interestingly, we observed that GluA1 and GluA2 also affected the cluster size of SD4 when co-expressed in COS cells. These results coincide with an increase in cluster size when stratified for co-localization with GluA1 or GluA2. We conclude that the cluster size of both SD4 and AMPAR puncta is significantly increased only when co-localized in COS cells, indicating a bi-directional interaction mechanism.

We were not able to identify a smaller domain of GluA1 or GluA2 sufficient for clustering with SD4. One possibility is that there are multiple regions within the AMPAR necessary for interacting with SD4. Pioneering work by Ben-Yaacov and colleagues using domain swaps demonstrated that AMPAR interaction with stargazin/TARP-γ2 primarily involves the AMPAR membrane domains M1 and M4 of neighboring subunits, with important contributions by the CT (Ben-Yaacov et al., 2017). Structural studies with cryo-EM support these functional results (Twomey et al., 2016; Zhao et al., 2016; Zhang et al., 2021). Attempts to express three constructs in COS cells were technically problematic; therefore, we could not pursue this approach. Since SD4 has been shown to affect the biophysical properties of GluA1 and GluA2-containing AMPARs (Matt et al., 2018), in future experiments we plan to continue the structure-function approach with electrophysiology to narrow down the critical domain. Moreover, the cryo-EM structures of native AMPARs indicates that SD4 is associated with AMPAR complexes that contain TARP-γ8 and CNIH-2 (Yu et al., 2021). Thus, it will be interesting to determine if SD4-dependent AMPAR clustering is influenced by the presence of TARP-γ8 and/or CNIH-2, or whether SD4 clusters bi-directionally with either of these two auxiliary factors. Furthermore, the co-expression of multiple auxiliary subunits might increase the efficiency of co-immunoprecipitation of AMPAR subunits observed with SD4 alone (Martin et al., 2021). It should be noted that clustering of AMPARs required the co-expression of stargazin/TARP-γ2 and PSD-95; stargazin/TARP-γ2 alone was not sufficient to change the distribution of AMPARs in heterologous cells (Chen et al., 2000). Thus, the mutually dependent clustering activity of SD4 with AMPARs might be unique to this auxiliary factor.

Intriguingly, the mutually dependent clustering requires incubation at 37^°^C, suggesting that endocytosis of surface labeled AMPARs is most likely necessary for clustering with SD4. In primary hippocampal neurons at steady-state most SD4 overlaps with endosomal markers (Martin et al., 2021) but some protein is available for surface labeling (Kirk et al., 2016; Martin et al., 2021). Thus, it is tempting to speculate that SD4 captures AMPARs at the plasma membrane for clustering *via* transport through an endocytic compartment. Current studies are addressing this possibility. Importantly, the increased cluster size of GluA1 that overlaps with SD4 is observed in primary hippocampal neurons upon chemical-LTP, suggesting that this clustering activity is a mechanism underlying the strengthening of synapses during synaptic plasticity. As the SD4-induced clustering of AMPARs likely occurs after endocytosis in heterologous cells, these intracellular clusters of AMPARs co-localized with SD4 are capable of being deployed to synapses upon chemical-LTP in hippocampal neurons. In addition, SD4 KO mice are deficient for LTD (Troyano-Rodriguez et al., 2019); thus, the intracellular clustering of AMPARs could also be employed during LTD as a mechanism to restrict the surface accumulation of AMPARs. We propose that SD4 establishes an intracellular pool of extrasynaptic AMPARs through the bidirectional clustering of SD4 and AMPARs necessary for regulating synaptic strength.

Our results demonstrate the effects of SD4 on clustering both GluA1 and GluA2 in heterologous cells. In SD4 KO mice, we observed significant reduction in both extrasynaptic GluA2 and extrasynaptic GluA1 puncta density (Matt et al., 2018), consistent with our observations in COS cells. As GluA1/2 heteromers constitute 95% of extrasynaptic AMPAR pool under baseline conditions (Lu et al., 2009), this suggests that SD4 is required to maintain extrasynaptic GluA1/2. Interestingly, certain effects of SD4 were specific for GluA1. For example, puncta size and intensity of both extrasynaptic and synaptic GluA1 (but not GluA2) were slightly reduced in SD4 KO neurons (Matt et al., 2018), indicating an additional role for SD4 in regulating GluA1. GluA1 homomers account for most, if not all, calcium-permeable AMPARs (CP-AMPARs) in hippocampus, which are largely absent at PSDs under basal conditions; however, under certain conditions, CP-AMPARs become transiently detectable at postsynaptic sites including the induction of LTP (Plant et al., 2006) and LTD (Sanderson et al., 2016). Thus, it is tempting to speculate that SD4 might establish reserve pools of GluA1 homomers that are transiently targeted to synapses during synaptic plasticity.

We were able to identify a minimal domain of GluA2 responsible for the interaction with the related protein SD1. SD4 and SD1 share approximately 35% overall amino acid sequence similarity, with higher similarity occurring within the membrane bound domain (Kalashnikova et al., 2010). However, while SD4 and SD1 share similarity only SD4 has been shown to affect the biophysical properties of GluA1 and GluA2-containing AMPARs (Lovero et al., 2013; Matt et al., 2018). The co-expression of GluA2 with SD1 shows the significant clustering of GluA2 when compared to GluA2 expression alone, which fits with previously observed results (Kalashnikova et al., 2010). Conversely, the co-expression of GluK2 with SD1 did not exhibit a clustering phenotype. Using the same GluK2/GluA2 chimeras, we observed the clustering of GluK2/A2/M1-3/S2/M4/CT chimera when co-expressed with SD1. In contrast to SD4, we observed clustering when SD1 was co-expressed with the GluK2/GluA2 chimera expressing only the M4 and CT domain of GluA2. We conclude that the minimal M4/CT domain of GluA2 is sufficient for clustering with SD1, which potentially explains the lack of SD1 effects on biophysical properties.

Most of the excitatory transmission in the brain is mediated by AMPA receptors. Furthermore, many neuropsychiatric and neurological disorders, such as Alzheimer’s disease and depression, can be characterized by abnormal AMPA receptor content and trafficking leading to impaired synapse function (Babaei, 2021; Ge and Wang, 2021; Kadriu et al., 2021). Therefore, understanding the complete mechanism behind AMPA receptor function is important for understanding disease. Continuing studies utilizing cultured hippocampal neurons and transgenic mouse models will be important to establish the role of SD4 trafficking to the synapse that may yield a better understanding of underlying mechanism behind neuropsychiatric and neurological disorders.

## Data Availability Statement

The raw data supporting the conclusions of this article will be made available by the authors, without undue reservation.

## Ethics Statement

The animal study was reviewed and approved by the UC Davis Institutional Animal Care and Use Committee (IACUC).

## Author Contributions

KP and ED: methodology and writing. KP, C-WH, and ED: investigation. KP, C-WH, HN, and ED: formal analysis. ED: funding acquisition and supervision. All authors contributed to the article and approved the submitted version.

## Conflict of Interest

The authors declare that the research was conducted in the absence of any commercial or financial relationships that could be construed as a potential conflict of interest.

## Publisher’s Note

All claims expressed in this article are solely those of the authors and do not necessarily represent those of their affiliated organizations, or those of the publisher, the editors and the reviewers. Any product that may be evaluated in this article, or claim that may be made by its manufacturer, is not guaranteed or endorsed by the publisher.
